# Photodegradation of Pollutants in the Hydrophobic Cores of Dissolved Organic Matter: When Is It Important?

**DOI:** 10.1021/acs.est.5c01331

**Published:** 2025-05-08

**Authors:** Elio Mondino, Luca Carena, Cheng Gu, Davide Vione

**Affiliations:** † Dipartimento di Chimica, 9314 Università degli Studi di Torino, Via Pietro Giuria 5, 10125 Torino, Italy; ‡ State Key Laboratory of Pollution Control and Resource Reuse, School of Environment, 12581 Nanjing University, Nanjing 210023, China

**Keywords:** hydrophobic contaminants, dissolved organic matter, photodegradation, phase partitioning, microheterogeneous distribution of ^•^OH and ^1^O_2_

## Abstract

In natural surface waters, hydroxyl radicals (^•^OH) and singlet oxygen (^1^O_2_) are known to occur not only in the bulk aqueous phase but also within the hydrophobic cores of dissolved organic matter (DOM). In these DOM sites, ^•^OH and ^1^O_2_ reach steady-state concentrations that are orders of magnitude higher than those in bulk water, which can enhance photodegradation of hydrophobic pollutants. In analogy with previous works, here, we use a two-phase reactivity model to address the importance of the phenomenon. The model requires much care to identify which variables are referred to the total solution volume (bulk water + DOM), the volume of the water bulk, and most importantly, that of the DOM phase. We suggest that DOM-phase partitioning could significantly affect the photodegradation of pollutants having an octanol–water partition coefficient log_10_ *K*_ow_ > 3. In the case of ^•^OH, differences should be expected between irradiation of solutions containing organic matter alone as the only ^•^OH source and irradiation of natural water samples where ^•^OH would also be generated by photolysis of nitrate and nitrite.

## Graphical abstract

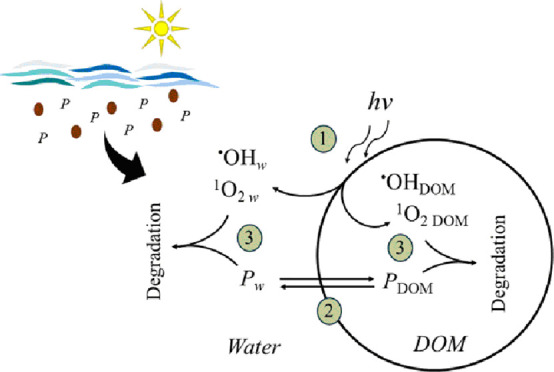

## 1 Introduction

Photochemical reactions play an important role in the attenuation of many contaminants in surface waters. Such reactions are usually divided into direct photolysis, where the pollutant gets transformed after it absorbs sunlight, and indirect photochemistry. In the latter case, sunlight is absorbed by naturally occurring compounds called photosensitizers that include, for instance, nitrate, nitrite, and chromophoric dissolved organic matter (CDOM).^1–3^ Illuminated photosensitizers generate transient species known as photochemically produced reactive intermediates (PPRIs) that include, among others, the hydroxyl (^•^OH) and carbonate (CO_3_^•^ ^–^) radicals, singlet oxygen (^1^O_2_), and CDOM triplet states (^3^CDOM*).^4,5^ PPRIs react with pollutants and cause their degradation; very often, a pollutant undergoes transformation by both direct photolysis and indirect photochemistry, and the prevailing reaction pathway(s) depend(s) upon both the molecule and the environmental conditions.^6,7^

Interestingly, PPRIs, such as ^•^OH, ^1^O_2_, as well as electrons generated by CDOM photoexcitation, show a microheterogeneous distribution within dissolved organic matter (DOM, not necessarily chromophoric).^8–12^ Therefore, in addition to occurring in the water bulk, these PPRIs are also found within hydrophobic DOM sites, where their steady-state concentrations are orders of magnitude higher than in water. Hydrophobic pollutants that partition significantly within DOM might thus undergo faster photooxidation (by ^•^OH and ^1^O_2_) and/or photoreduction (by e^–^) compared to hydrophilic compounds that are only or prevalently degraded in the water bulk (see Figure 1).

**Figure 1:**
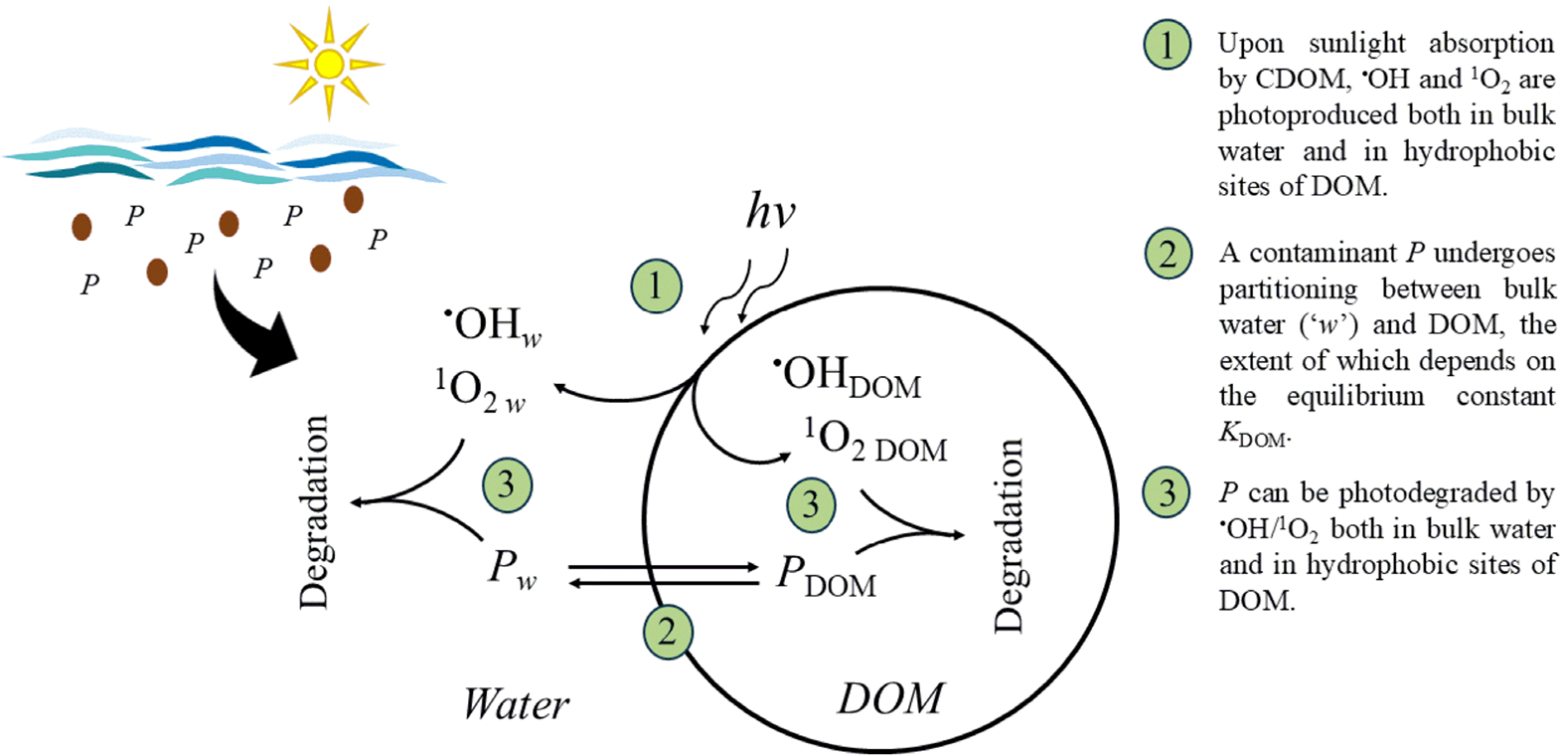
Scheme showing the partitioning and photoreactivity of a hydrophobic contaminant *P* between bulk water and hydrophobic DOM sites.

In the case of ^1^O_2_, the reason for its high occurrence within DOM could be the absence of the main ^1^O_2_ quencher (H_2_O) in the hydrophobic DOM cores, a circumstance that would increase the ^1^O_2_ lifetime and, as a consequence, its steady-state concentration.^8^ In the case of ^•^OH, formation pathways by photolysis of CDOM-hydroxylated derivatives^13^ and the physical closeness between precursors/photosensitizers and hydrophobic pollutants could possibly play a role.

Despite the high importance of the above findings, we feel that an obstacle to their routine application in studies aimed at elucidating the photochemical fate of contaminants is that it is not yet totally clear which pollutants are significantly transformed by ^•^OH and/or ^1^O_2_ within DOM cores. The present contribution has the purpose of better elucidating this issue, to foresee which compounds may be affected and to what extent, in order to better highlight the environmental importance of the microheterogeneous distribution of ^•^OH and ^1^O_2_ within the DOM.

## 2 Water–DOM Partitioning Equilibria and Reference Volumes

The microheterogeneous distribution of ^•^OH and ^1^O_2_ within the DOM can be important if pollutants undergo partitioning between the water phase and DOM hydrophobic cores. Partitioning can be described by equilibria that bear analogy with the formation of complexes in water.^8,9^ When a pollutant (*P*) in water is assumed, which undergoes partitioning equilibrium with DOM (reaction 1)­

$$P_{w}+\mathrm{DOM}⇆P_{\mathrm{DOM}}$$ 1

where *P*_w_ is the pollutant in the water bulk and *P*_DOM_ is the pollutant within DOM. Reaction 1 is described by the partitioning constant *K*_DOM_ ^8,9^

$$K_{\mathrm{DOM}}=\frac{{\left[P_{\mathrm{DOM}}\right]}_{w}}{{\left[P_{w}\right]}_{w}{\left[\mathrm{DOM}\right]}_{w}}=10^{6}{\mathrm{mg}}_{C}{\left({\mathrm{kg}}_{C}\right)}^{-1}\frac{{\left[P_{\mathrm{DOM}}\right]}_{w}}{\mathrm{DOC}{\left[P_{w}\right]}_{w}}$$ 2

where DOC is the dissolved organic carbon. Some important considerations are warranted about eq 2: (i) The concentration values should be referred to the total solution volume *V*_tot_ = *V*_w_ + *V*_DOM_, where *V*_w_ is the volume of the water bulk and *V*_DOM_ is the volume of the DOM phase; however, because *V*_w_ ≫ *V*_DOM_, it is *V*_tot_ ≈ *V*_w_; (ii) [DOM]_w_ is the concentration of DOM in water expressed in (kg_C_ L^–1^) units; thus, [DOM]_w_ = 10^–6^ kg_C_ (mg_C_)^−1^ of DOC if we express DOC in the usual (mg_C_ L^–1^) units; (iii) [*P*_w_]_w_ is the concentration of the pollutant in water, referred to the volume *V*_tot_ ≈ *V*_w_; (iv) [*P*_DOM_]_w_ is the concentration of *P* that is incorporated into the DOM phase, but the volume is referred to *V*_tot_ ≈ *V*_w_. Therefore, [*P*_DOM_]_w_ is the number of moles of *P* in the DOM phase divided by the water volume.

The reference volume is important because, when speaking about microheterogeneous distributions within DOM, the steady-state concentration values [^•^OH_DOM_] and [^1^O_2 DOM_] are typically referred to *V*_DOM_.^8,9^ In other words, [^•^OH_DOM_] and [^1^O_2 DOM_] should be intended as moles of ^•^OH (or ^1^O_2_) confined within DOM, divided by *V*_DOM_ (hereafter, they will be indicated as [^•^OH_DOM_]_DOM_ and [^1^O_2 DOM_]_DOM_, respectively). In contrast, [^•^OH_w_]_w_ and [^1^O_2 w_]_w_ represent the moles of ^•^OH (or ^1^O_2_) occurring in the water bulk, divided by *V*_tot_ ≈ *V*_w_. This distinction plays a key role when comparing the photoreaction rates of a pollutant *P* within DOM and in the water bulk.

Also note that DOM has a density of approximately 1.5 g cm^–3^ and that the mass fraction of carbon in DOM is approximately 0.5.^8^ On this basis, it is *V*_DOM_ ≈ (7.5 × 10^5^)^−1^ DOC *V*_w_.

## 3 Reaction Kinetics

Text S1 of the Supporting Information describes the procedure by which it is possible to assess which fraction of the pollutant *P* is degraded in the water bulk and which fraction is in the DOM phase. The following equations are obtained for both ^•^OH and ^1^O_2_:

γOH•=10−6KDOMDOC[OHDOM•]DOM[OHw•]w 3

γO21=10−6KDOMDOC[O2⁡DOM1]DOM[O12⁡w]w 4

where γ_^•^OH_ is the ratio between the ^•^OH reaction rates in DOM and water, both referred to the same volume *V*_w_ (γ_^•^OH_ = *R*_w*P*,DOM_/*R*_*P*,w_). Therefore, the reaction between *P* and ^•^OH would mainly take place within DOM when γ_^•^OH_ > 1 and in the water bulk when γ_^•^OH_ < 1. Similar issues hold for γ_^1^O_2__ and ^1^O_2_.

A shortcoming with eqs 3 and 4 is that *K*_DOM_ is known for only a few pollutants, differently from the octanol–water partition coefficient *K*_ow_ for which much more widespread information is available. For this reason, we exploited a correlation between *K*_DOM_ and *K*_ow_ (*K*_DOM_ ≈ 0.26*K*_ow_).^14^ By replacement of *K*_DOM_ with 0.26*K*_ow_, eqs 3 and 4 are modified into eqs S5 and S7 of the Supporting Information.

We made a number of assumptions, the justifications of which are given later (section 4). First of all, we assumed that [^•^OH_DOM_]_DOM_ ([^•^OH_w_]_w_)^−1^ = 10^2^. Moreover, in the presence of CDOM alone in solution, [^•^OH_w_]_w_ would be about constant with varying DOC (Figure S2 of the Supporting Information); the same applies to [^•^OH_DOM_]_DOM_. Finally, we assumed [^1^O_2 DOM_]_DOM_ = 2 × 10^–12^ M.

The values of [^1^O_2 w_]_w_ (eq 4) were assessed by photochemical modeling, for which we used the Aquatic Photochemistry of Environmental Xenobiotics (APEX) software. APEX predicts pseudo-first-order rate coefficients of pollutant transformation and steady-state PPRI concentrations in the aqueous phase, including [^•^OH_w_]_w_ and [^1^O_2 w_]_w_, as a function of photolysis quantum yields, second-order reaction rate constants, absorption spectra, water depth, and water chemistry parameters, such as nitrate, nitrite, DOC, carbonate, and bicarbonate.^15^ Irradiation experiments to describe the microheterogeneous distribution of ^•^OH and ^1^O_2_ within DOM have been carried out in the laboratory^8,9,16^ for relatively short (centimeter-scale) values of the optical path length that corresponds to water depth in the APEX model. For this reason, we used a low depth of *d* = 5 cm in APEX simulations. Note that, when water depth is low enough that the whole aqueous phase is fully illuminated, reaction kinetics and steady-state concentrations become depth-independent.^15^ APEX yielded here the values of [^1^O_2 w_]_w_ as a function of the DOC. For additional details into the use of APEX, see Text S2 of the Supporting Information.

The trends of γ vs DOC are shown in Figure 2 for different values of log_10_ *K*_ow_ (0–8) and, specifically, γ_^•^OH_ [eq 3 (eq S5) and Figure 2a] and γ_^1^O_2__ [eq 4 (eq S7) and Figure 2b]. Known values of log_10_ *K*_ow_ for sample pollutants are also reported on the plots for comparison/reference. It can be seen from Figure 2 that DOM-phase transformation (γ > 1) is understandably enhanced at high DOC in the case of ^•^OH, and that reactions within DOM can become important if log_10_ *K*_ow_ > 3. In contrast, if log_10_ *K*_ow_ < 3, DOM-phase reactions would hardly be able to prevail over water–bulk processes.

**Figure 2:**
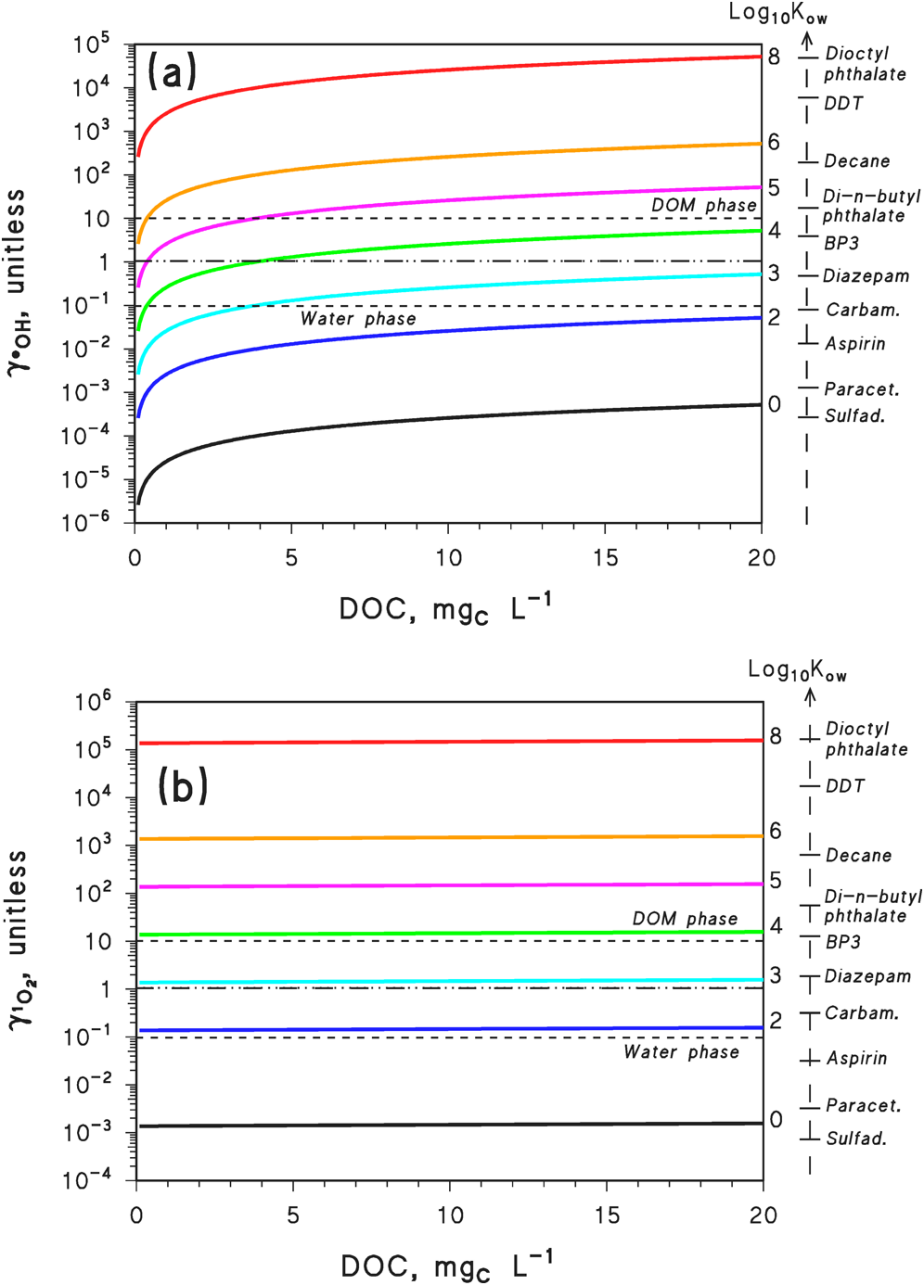
Trends of γ vs DOC, based on eq 3 (eq S5) (^•^OH, a) and eq 4 (eq S7) (^1^O_2_, b), for different values of log_10_ *K*_ow_ (the latter values are reported on the right *y* axis, near each relevant curve; note that *K*_DOM_ ≈ 0.26*K*_ow_). DOM-phase reactions strongly prevail for γ > 10, and water-phase reactions strongly prevail for γ < 0.1. The dash-dotted horizontal lines refer to γ = 1, while the dashed horizontal lines refer to γ = 0.1 and to γ = 10. It was assumed that [^•^OH_DOM_]_DOM_ ([^•^OH_w_]_w_)^−1^ = 10^2^ and [^1^O_2 DOM_]_DOM_ = 2 × 10^–12^ M. The log_10_ *K*_ow_ values of sample compounds (see Table S1 of the Supporting Information for references) are reported alongside the right *y* axis and correspond to the numerical values reported to the left of the vertical arrow. “Paracet.”, paracetamol; “Sulfad.”, sulfadiazine; “Carbam.”, carbamazepine; and “BP3”, benzophenone 3. Note that, for compounds undergoing acid–base equilibria, the log_10_ *K*_ow_ value refers to the neutral form.

By comparison, Table S1 of the Supporting Information reports the log_10_ *K*_ow_ values of several compounds. It is shown in the table that the condition log_10_ *K*_ow_ > 3 includes different classes of pollutants, such as pesticides, plasticizers, ultraviolet (UV) filters, pharmaceuticals, fragrances, and disinfectants.

## 4 Pollutant Degradation Kinetics as a Function of DOC and *K*_ow_

The overall degradation rate of *P* in the whole solution, which includes both the water bulk and the DOM phase, is *R*_*P*,tot_ = *R*_*P*,w_ + *R*_w*P*,DOM_. The same rate can also be expressed as *R*_*P*,tot_ = *k*_*P*,tot_^′^[*P*_tot_], where *k*_*P*,tot_^′^ is the pseudo-first-order rate coefficient of *P* degradation and [*P*_tot_] = [*P*_DOM_]_w_ + [*P*_w_]_w_. By considering eqs S1–S4 of the Supporting Information, in the case of the reaction with ^•^OH, one gets the following:

(kP,tot′)OH•=kw,P,OH•[OHw•]w+10−6KDOMDOC[OHDOM•]DOM1+10−6KDOMDOC 5

Equation 5 is the general equation that describes *P* degradation by ^•^OH in an aquatic system that contains DOM. If *K*_DOM_ → 0 and/or DOC → 0, the equation reduces to (*k*_*P*,tot_^′^)_^•^OH_ = *k*_w,*P*,^•^OH_[^•^OH_w_]_w_ that is the typical pseudo-first-order expression of *P* degradation by ^•^OH in a homogeneous aqueous solution, where *k*_w,*P*,^•^OH_ is the second-order reaction rate constant between *P* and ^•^OH. A corresponding equation is obtained for (*k*_*P*,tot_^′^)_^1^O_2__, too, where *k*_w,*P*,^•^OH_, [^•^OH_w_]_w_, and [^•^OH_DOM_]_DOM_ are replaced by the corresponding expressions relevant to ^1^O_2_. Note that [^•^OH_DOM_]_DOM_ and [^1^O_2 DOM_]_DOM_ are referred to the DOM phase (*V*_DOM_) and are independent of how much DOM is present in solution.^8,9,16^

Equation 5 allows for an assessment of (*k*_*P*,tot_^′^)_^•^OH_ vs DOC. To compute (*k*_*P*,tot_^′^)_^•^OH_, one needs the trend of [^•^OH_w_]_w_ vs DOC, which has to take into account the fact that CDOM produces ^•^OH, while DOM scavenges it.^5,17^ We derived such a trend by using the APEX software (see also Text S2 of the Supporting Information).

An issue with eq 5 is that it depends upon *k*_w,*P*,^•^OH_, which would differ for different contaminants. To obtain a more general equation, we considered the ratio θ_^•^OH_ between (*k*_*P*,tot_^′^)_^•^OH_ and $\underset{\mathrm{DOC}\to 0}{\lim}$ ­(*k*_*P*,tot_^′^)_^•^OH_ as follows (an analogous expression is obtained for ^1^O_2_; see equation S21 of the Supporting Information):

θOH•=(1[OHw•]w)DOC→0[OHw•]w+10−6KDOMDOC[OHDOM•]DOM1+10−6KDOMDOC 6

The results for ^•^OH are shown in Figure 3, which plots θ_^•^OH_ as a function of the DOC for different values of log_10_ *K*_ow_ (as done in the previous section, we used the correlation *K*_DOM_ ≈ 0.26*K*_ow_). In the case of Figure 3a, the results are relevant to a system that contains only DOM/CDOM and has no nitrate, nitrite, or inorganic carbon, as for the irradiation experiments reported in the literature.^9^ When log_10_ *K*_ow_ < 3, the reaction would almost exclusively take place in the water phase and the reported DOC trend of θ_^•^OH_ reflects that of [^•^OH_w_]_w_.

**Figure 3:**
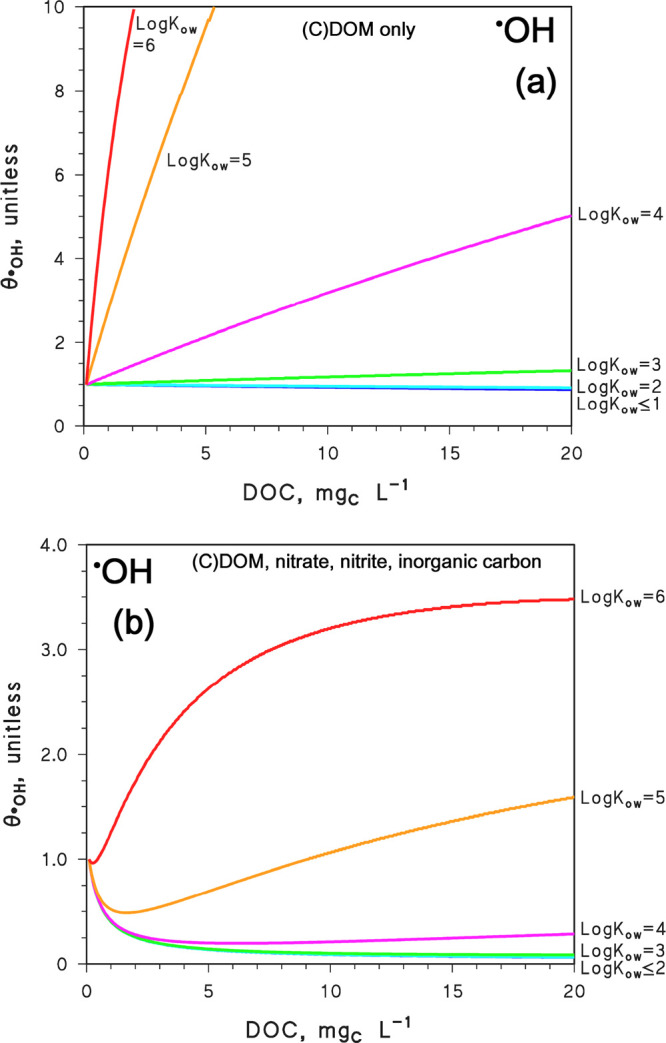
Trends of θ_^•^OH_ vs DOC, for different values of log_10_ *K*_ow_. Water conditions: (a) 5 cm depth, absence of NO_3_^–^, NO_2_^–^, HCO_3_^–^, and CO_3_^2–^, as per irradiation experiments in the presence of organic matter alone; (b) 5 cm depth, 10^–4^ M NO_3_^–^, 10^–6^ M NO_2_^–^, 10^–3^ M HCO_3_^–^, and 10^–5^ M CO_3_^2–^, which could represent irradiation of an actual water sample. The θ_^•^OH_ trends are based on eq 6, and only [^•^OH_w_]_w_ (modeled with APEX) differs in the two cases.

In the presence of CDOM alone (Figure 3a), if log_10_ *K*_ow_ < 3, there is only a limited dependence of θ_^•^OH_ on the DOC and log_10_ *K*_ow_. In such a case, the aqueous-phase reaction would prevail, where ^•^OH_w_ would only be photogenerated by CDOM and scavenged by DOM. As a consequence, [^•^OH_w_]_w_ would change very little with varying DOC (see Figure S2 of the Supporting Information), which justifies one of the assumptions made in section 3. Some slight enhancement in photodegradation kinetics is apparent for log_10_ *K*_ow_ = 3, and more marked effects can be seen if log_10_ *K*_ow_ ≥ 4.

An alternative way to look at eq 5 would be to consider an apparent steady-state ^•^OH concentration, (*k*_*P*,tot_^′^)_^•^OH_ = *k*_w,*P*,^•^OH_[^•^OH]_app_, as done for instance by Yan et al.^9^ By considering that, from eqs 5 and 6, it is [^•^OH]_app_ = θ_^•^OH_ {limDOC→0[OHw•]w} , it is quite easy to translate the [^•^OH]_app_ formalism into the θ_^•^OH_ formalism. By so doing, it is possible to fit available experimental data^9^ with eq 6, with [^•^OH_DOM_]_DOM_ ([^•^OH_w_]_w_)^−1^ as the fit parameter. The results are reported in Figure S3 of the Supporting Information; very reasonable agreement between eq 6 and the experimental data could be obtained with [^•^OH_DOM_]_DOM_ ([^•^OH_w_]_w_)^−1^ = 10^2^, which is an *a posteriori* justification of previous assumptions.

The scenario of Figure 3a is relevant to experiments where humic substances are irradiated in ultrapure water, while natural water samples also contain other ^•^OH sources, such as nitrate and nitrite.^4,6^ Results that may be relevant to the irradiation of natural water samples are listed in Figure 3b. In this case, the aqueous-phase reaction (always important if log_10_ *K*_ow_ < 3) undergoes strong inhibition at high DOC, because ^•^OH_w_ photogenerated by nitrate and nitrite would be largely scavenged by DOM and, to a lesser extent, by inorganic carbon ([^•^OH_w_]_w_ decreases with increasing DOC; see Figure S2 of the Supporting Information). As a consequence, different from the case of Figure 3a, degradation kinetics for DOC = 20 mg_C_ L^–1^ is predicted to be much slower than for DOC → 0 mg_C_ L^–1^ if log_10_ *K*_ow_ < 3. The trend does not change much if log_10_ *K*_ow_ = 3 or 4, despite some acceleration of the reaction at high DOC compared to lower log_10_ *K*_ow_ values. In the case of log_10_ *K*_ow_ = 5, the inhibition of the ^•^OH_w_ process with increasing DOC is soon offset by the growing importance of the ^•^OH_DOM_ reaction. Acceleration of the process with increasing DOC is even more evident when log_10_ *K*_ow_ = 6 (or higher; data not shown).

Analogous reasoning can be carried out for the ^1^O_2_ reactions. In this case, good agreement between experimental [^1^O_2_]_app_ values^16^ and model predictions (eq S20 of the Supporting Information) could be obtained with [^1^O_2 DOM_]_DOM_ ∼ 2 × 10^–12^ M (see Figure S4 of the Supporting Information), which justifies the last assumption made in section 3. As far as θ_^1^O_2__ is concerned (eq S21 of the Supporting Information), it is predicted to increase with increasing DOC. If log_10_ *K*_ow_ ≤ 2, the θ_^1^O_2__ trend reflects that of [^1^O_2 w_]_w_ irrespective of the actual log_10_ *K*_ow_ value, because the water–bulk reaction prevails over the DOM-phase reaction. Interestingly, [^1^O_2 w_]_w_ increases with increasing DOC (see Figure S2 of the Supporting Information), because ^1^O_2_ is photogenerated by CDOM as its only source and its quenching (collision with the water solvent) is independent of organic matter.^4,5^ The reaction kinetics maintain the same linear increase with increasing DOC, but they get slightly faster if log_10_ *K*_ow_ = 3 and considerably faster if log_10_ *K*_ow_ > 3 (see Figure S5 of the Supporting Information).

## 5 Environmental Implications

The microheterogeneous distribution of ^•^OH and ^1^O_2_ within the hydrophobic cores of DOM has important implications for the photodegradation of hydrophobic pollutants. When dealing with a two-phase system (water bulk and DOM phase), care should be taken with the equations that describe the reaction kinetics. In particular, the reference volume (*V*_DOM_ vs *V*_w_ ≈ *V*_tot_) has to be clearly identified for each relevant quantity, because the steady-state concentrations of ^•^OH_DOM_ and ^1^O_2 DOM_ are typically referred to *V*_DOM_, while the other quantities are best referred to *V*_tot_ or *V*_w_.

Here, we show that DOM-phase reactions can be important for compounds having log_10_ *K*_ow_ > 3. The effect of reactions with ^•^OH_DOM_ and ^1^O_2 DOM_ is a degradation enhancement with increasing DOC and/or log_10_ *K*_ow_. In the case of ^•^OH, there is a difference in the results of irradiation of organic matter solutions vs natural water samples, as the latter may contain additional photochemical sources of hydroxyl radicals, such as nitrate and nitrite. With organic matter alone, degradation kinetics is expected to increase with DOC if log_10_ *K*_ow_ > 3. In the case of natural water samples, ^•^OH_w_ photogenerated by nitrate and nitrite would be largely scavenged by DOM, and the DOM-phase reactions involving ^•^OH_DOM_ might not be sufficient to overcome the scavenging of ^•^OH_w_ by DOM itself. Compared to a pure water-phase reaction, the overall effect would be a lower inhibition of degradation with increasing DOC if log_10_ *K*_ow_ < 5, a more balanced offset if log_10_ *K*_ow_ = 5, and degradation enhancement for higher log_10_ *K*_ow_ values.

Despite these differences, DOM-phase reactions could significantly affect the lifetimes of the relevant contaminants in all scenarios and should thus not be ignored in the case of compounds that are sufficiently hydrophobic.

Finally, ^1^O_2_ is generated by CDOM triplet states (^3^CDOM*), and the interactions of substrates with (C)­DOM is known to enhance triplet-sensitized degradation.^18^ Therefore, for hydrophobic compounds that partition within organic matter, there could be the potential to undergo triplet sensitization in addition to reactions with ^•^OH and ^1^O_2_ in the DOM phase.

## Supplementary Material



## Notes

Derivation of eqs 3 and 4, description of the APEX software, compilation of Log_10_K_ow_ values, comparison between model predictions and available experimental data, and model results in the case of ^1^O_2_ (PDF)
