# Supplementary material for: Photodegradation of Pollutants in the Hydrophobic Cores of Dissolved Organic Matter: When Is It Important?
Source: Environ Sci Technol. 2025 May 8;59(19):9369–74. doi: 10.1021/acs.est.5c01331 (PMC12096434; doi:10.1021/acs.est.5c01331)
Supplement: Supplementary file 1 [file es5c01331_si_001.pdf]

SUPPORTING INFORMATION

Photodegradation of Pollutants in the Hydrophobic Cores of Dissolved Organic Matter: When is it Important?

Elio Mondino,[a] Luca Carena,[a] Cheng Gu,[b] Davide Vione [a]*

[a] Università degli Studi di Torino, Dipartimento di Chimica, Via Pietro Giuria 5, 10125 Torino, Italy.

[b] State Key Laboratory of Pollution Control and Resource Reuse, School of Environment, Nanjing University, Nanjing 210023, China.

* Corresponding author. E-mail: *davide.vione@unito.it*

Summary: 18 pages, 5 figures, 1 table.

Contents

Test S1. Degradation kinetics in the DOM phase vs. the water bulk	Page S2
Text S2. Use of the APEX software for the assessment of photochemical reactions in surface freshwaters	Page S4
Figure S1	Page S5
Figure S2	Page S7
Table S1	Page S8
Figure S3 & Equations (S17,S18)	Page S12
Figure S4 & Equation (S19)	Page S13
Figure S5 & Equations (S20,S21)	Page S14
References	Page S15

Text S1. Degradation kinetics in the DOM phase vs. the water bulk

Assume the case of P degradation by $^{\bullet}$OH. The expression for the reaction rate of P in the water bulk is rather straightforward:

$$R_{P,w} = k_{w,P,^{\bullet}\mathrm{OH}}\,[^{\bullet}\mathrm{OH}_w]_w\,[P_w]_w \qquad \text{(S1)}$$

Where $k_{w,P,^{\bullet}\mathrm{OH}}$ (units of M^{-1} s^{-1}) is the second-order rate constant of the water-bulk reaction between P_w and $^{\bullet}OH_w$. The molar concentrations refer to species in the aqueous phase. It is possible to define a similar reaction rate for P with $^{\bullet}$OH in the the DOM phase, $R_{P,\mathrm{DOM}}$:

$$R_{P,\mathrm{DOM}} = k_{\mathrm{DOM},P,^{\bullet}\mathrm{OH}}\,[^{\bullet}\mathrm{OH}_{\mathrm{DOM}}]_{\mathrm{DOM}}\,[P_{\mathrm{DOM}}]_{\mathrm{DOM}} \qquad \text{(S2)}$$

Where $k_{\mathrm{DOM},P,^{\bullet}\mathrm{OH}}$ (units of M^{-1} s^{-1}) is the second-order rate constant of the DOM-phase reaction between P_{DOM} and $^{\bullet}OH_{\mathrm{DOM}}$, and the molar concentrations refer to species in the DOM phase.

The reaction rates $R_{P,w}$ and $R_{P,\mathrm{DOM}}$ cannot be directly compared, because the former is referred to the water bulk (V_w) and the latter to the DOM phase (V_{DOM}). To enable comparison, and to assess which fraction of P reacts with $^{\bullet}OH_w$ in the bulk and which reacts with $^{\bullet}OH_{\mathrm{DOM}}$ within DOM, it is possible to define $R_{wP,\mathrm{DOM}}$ as the reaction rate between P_{DOM} and $^{\bullet}OH_{\mathrm{DOM}}$, but referred to the volume of the solution and not to the volume of DOM. In this way, comparison between $R_{wP,\mathrm{DOM}}$ and $R_{P,w}$ is possible. $R_{wP,\mathrm{DOM}}$ would be expressed as $R_{wP,\mathrm{DOM}} = R_{P,\mathrm{DOM}}\,V_{\mathrm{DOM}}\,(V_w)^{-1}$ and, therefore:

$$R_{wP,\mathrm{DOM}} = k_{\mathrm{DOM},P,^{\bullet}\mathrm{OH}}\,[^{\bullet}\mathrm{OH}_{\mathrm{DOM}}]_{\mathrm{DOM}}\,[P_{\mathrm{DOM}}]_w \qquad \text{(S3)}$$

Where $[P_{\mathrm{DOM}}]_w = [P_{\mathrm{DOM}}]_{\mathrm{DOM}}\,V_{\mathrm{DOM}}\,(V_w)^{-1}$. Note that, from the partitioning equilibrium of P between the water and DOM phases (main manuscript, section 2), it is $[P_{\mathrm{DOM}}]_w = 10^{-6}\,K_{\mathrm{DOM}}\,\mathrm{DOC}\,[P_w]_w$. It is thus possible to calculate the ratio $\gamma_{^{\bullet}\mathrm{OH}} = R_{wP,\mathrm{DOM}} / R_{P,w}$ between the reaction rates in DOM and water, both referred to the same volume V_w. When deriving $\gamma_{^{\bullet}\mathrm{OH}}$ we make the reasonable hypothesis that $k_{\mathrm{DOM},P,^{\bullet}\mathrm{OH}} = k_{w,P,^{\bullet}\mathrm{OH}}$,[1] obtaining the following expression:

$$\gamma_{^{\bullet}\mathrm{OH}} = 10^{-6} K_{\mathrm{DOM}}\,\mathrm{DOC}\,\frac{[^{\bullet}\mathrm{OH}_{\mathrm{DOM}}]_{\mathrm{DOM}}}{[^{\bullet}\mathrm{OH}_w]_w} \qquad \text{(S4)}$$

The reaction between P and $^{\bullet}$OH would mainly take place within DOM when $\gamma_{^{\bullet}\mathrm{OH}} > 1$, and in the water bulk when $\gamma_{^{\bullet}\mathrm{OH}} < 1$.

The main issue with **equation (S4)** is its reliance on K_{DOM}, which is not the most widely used partitioning constant for pollutants and would often be unavailable. However, pollutants undergoing effective DOM partitioning are hydrophobic compounds, and for most of them the octanol-water partition coefficient K_{ow} is known. Several correlation studies between K_{DOM} and K_{ow} have been

carried out, and an average of the results is $\mathrm{Log}_{10}\ K_{DOM}$ = (1.00±0.14) $\mathrm{Log}_{10}\ K_{ow}$ – (0.58±0.17).[2] From this relationship one gets $K_{DOM} \approx 0.26\ K_{ow}$, which can be introduced in **equation (S4)** to obtain **equation (S5)**:

$$\gamma_{^{\bullet}\mathrm{OH}} = 2.6\times10^{-7}\, K_{ow}\ \mathrm{DOC}\frac{[^{\bullet}\mathrm{OH}_{\mathrm{DOM}}]_{\mathrm{DOM}}}{[^{\bullet}\mathrm{OH}_{w}]_{w}} \qquad \text{(S5)}$$

Analogous reasoning can be carried out for the reaction between P and $^1O_2$, to obtain the following:

$$\gamma_{^1\mathrm{O}_2} = 1\times10^{-6}\, K_{\mathrm{DOM}}\ \mathrm{DOC}\frac{[^1\mathrm{O}_{2\ \mathrm{DOM}}]_{\mathrm{DOM}}}{[^1\mathrm{O}_{2\ w}]_{w}} \qquad \text{(S6)}$$

$$\gamma_{^1\mathrm{O}_2} = 2.6\times10^{-7}\, K_{ow}\ \mathrm{DOC}\frac{[^1\mathrm{O}_{2\ \mathrm{DOM}}]_{\mathrm{DOM}}}{[^1\mathrm{O}_{2\ w}]_{w}} \qquad \text{(S7)}$$

Text S2. Use of the APEX software for the assessment of photochemical reactions in surface freshwaters

APEX (Aquatic Photochemistry of Environmental Xenobiotics) is a software tool that computes aquatic photoreaction kinetics as a function of water chemistry and depth.[3] The photochemistry model behind APEX and, especially, behind the calculation by APEX of the steady-state [$^{\bullet}$OH] and [$^1O_2$] in the bulk aqueous phase is here described in detail.

Water absorption spectrum

A correlation has been found between the absorption spectra of natural water samples and their content of dissolved organic matter (DOM), expressed as the DOC (units of mg_C L^{-1}). The following equation holds for the water spectrum, with a σ-level uncertainty of approximately 20%:[4]

$$A_1(\lambda) = 0.45 \cdot DOC \cdot e^{-0.015\,\lambda} \qquad \text{(S8)}$$

Where $A_1(\lambda)$ [cm^{-1}] is the specific absorbance of lake water, namely the absorbance over an optical path length b = 1 cm. From the values of the absorbance, it is possible to assess the photon flux absorbed by water, as follows:

$$P_{a,w} = \int_{\lambda} p°(\lambda)\ (1-10^{-A_1(\lambda)})\, d\lambda \qquad \text{(S9)}$$

where $p°(\lambda)$ is the spectral photon flux density of sunlight (see **Figure S1** for its trend over wavelength). Note that sunlight absorption by natural waters between 280 and 500 nm is almost exclusively accounted for by CDOM, thus $P_{a,w} \sim P_{a,\mathrm{CDOM}}$.

Formation and scavenging of hydroxyl radicals, $^{\bullet}$OH

CDOM (chromophoric dissolved organic matter), nitrite, and nitrate are the main $^{\bullet}$OH sources in surface waters. The exact pathway(s) for the production of $^{\bullet}$OH by CDOM is/are still under debate, but they should involve light-excited CDOM at some level.[5,6]

$$NO_3^- + h\nu + H^+ \rightarrow {}^{\bullet}OH + {}^{\bullet}NO_2 \qquad \text{(S10)}$$

$$NO_2^- + h\nu + H^+ \rightarrow {}^{\bullet}OH + {}^{\bullet}NO \qquad \text{(S11)}$$

$$\mathrm{CDOM} + h\nu \rightarrow {}^{\bullet}OH \qquad \text{(S12)}$$

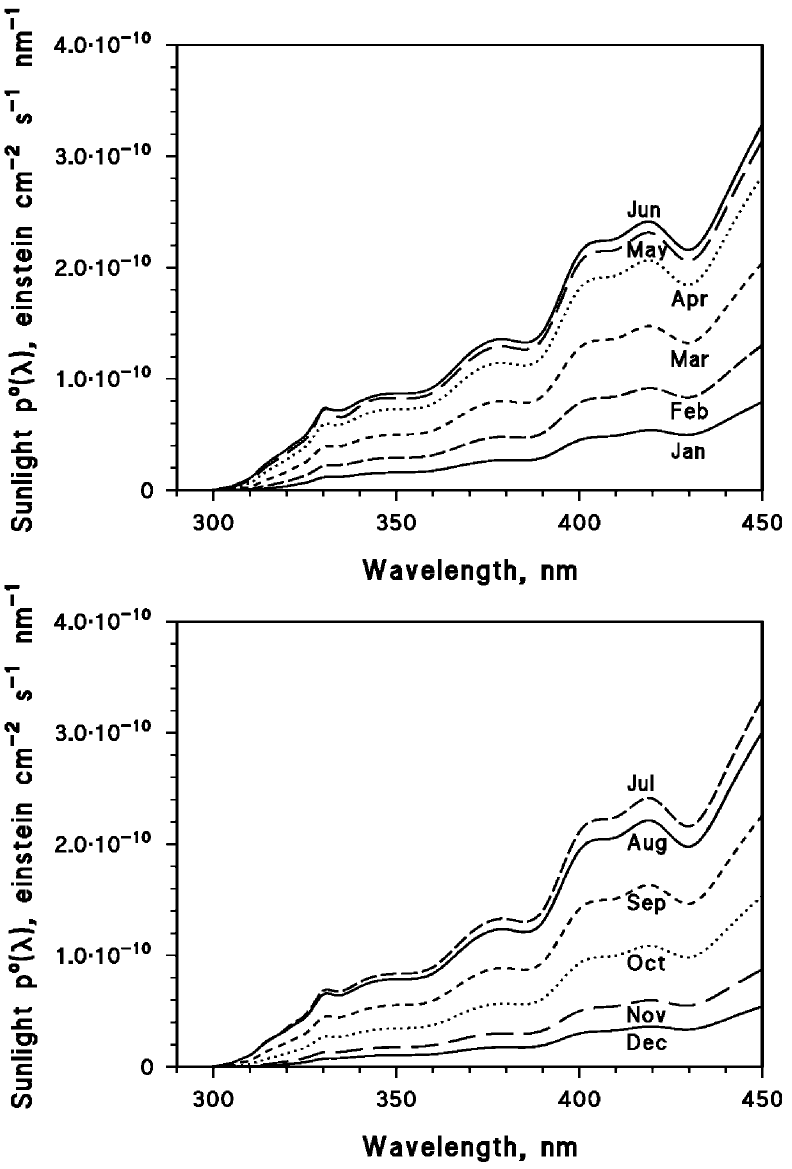

Figure S1. Spectral photon flux density of sunlight ($p°(\lambda)$) in the different months of the year (referred to the 15th day of each month). The data are referred to cloudless sky and are an 8-h average from 8 am to 4 pm.[7]

The rate of $^{\bullet}$OH photogeneration by nitrate, nitrite, and CDOM depends on the respective absorbed photon fluxes and on the quantum yields Φ of $^{\bullet}$OH production:[3]

$$R_{^{\bullet}OH,NO_3^-} = \int_{\lambda} \left[p°(\lambda)\ \Phi_{^{\bullet}OH,NO_3^-} \frac{\varepsilon_{NO_3^-}(\lambda)[NO_3^-]}{A_1(\lambda)} (1-10^{-A_1(\lambda)}) \right] d\lambda \qquad (S13)$$

$$R_{^{\bullet}OH,NO_2^-} = \int_{\lambda} \left[p°(\lambda)\ \Phi_{^{\bullet}OH,NO_2^-}(\lambda) \frac{\varepsilon_{NO_2^-}(\lambda)[NO_2^-]}{A_1(\lambda)} (1-10^{-A_1(\lambda)}) \right] d\lambda \qquad (S14)$$

$$R_{^{\bullet}OH,CDOM} = \int_{\lambda} \left[p^{\circ}(\lambda)\ \Phi_{^{\bullet}OH,CDOM} (1-10^{-A_1(\lambda)}) \right]\ d\lambda \qquad (S15)$$

where $\varepsilon_{NO_3^-}(\lambda)$ and $\varepsilon_{NO_2^-}(\lambda)$ are the molar absorption coefficients of nitrate and nitrite, respectively, while $[NO_3^-]$ and $[NO_2^-]$ are the respective molar concentrations. Furthermore, $\Phi_{^{\bullet}OH,NO_3^-} = 0.01$, $\Phi_{^{\bullet}OH,CDOM} \sim 3\times10^{-5}$, and $\Phi_{^{\bullet}OH,NO_2^-}(\lambda)$ are the quantum yields of $^{\bullet}OH$ photoproduction. In the case of nitrate and nitrite, the relevant equations for the $^{\bullet}OH$ production rate take into account competition with CDOM for sunlight irradiance. The same is not necessary for CDOM, which absorbs almost the totality of incoming sunlight below 500 nm.

$\Phi_{^{\bullet}OH,NO_2^-}(\lambda)$ varies with λ[nm], from 0.068 below 300 nm to 0.025 above 350 nm. APEX uses discrete numerical values of $\Phi_{^{\bullet}OH,NO_2^-}(\lambda)$, but an approximated (empirical) functional form would read as follows: $\Phi_{^{\bullet}OH,NO_2^-}(\lambda) \sim 0.042\ [10^{320-\lambda}\ (10^{320-\lambda} + 1.7\times10^5)^{-1}]^{0.033} + 0.025$. Once the $^{\bullet}OH$ formation rates are computed separately, the overall rate can be calculated as: $R_{^{\bullet}OH,tot} = R_{^{\bullet}OH,NO_3^-} + R_{^{\bullet}OH,NO_2^-} + R_{^{\bullet}OH,CDOM}$. In addition to being photogenerated, $^{\bullet}OH$ is scavenged by natural water components, with scavenging rate constant $\Sigma_i\ k_{Si}\ [S_i] = 5\times10^4\ DOC + 8.5\times10^6\ [HCO_3^-] + 3.9\times10^8\ [CO_3^{2-}] + 1.1\times10^{10}\ [Br^-] + 1.0\times10^{10}\ [NO_2^-]\ s^{-1}$, where the DOC [$mg_C\ L^{-1}$] is the dissolved organic carbon. On this basis, the calculation of the steady-state $^{\bullet}OH$ in the water bulk is carried out as follows: $[^{\bullet}OH_w]_w = R_{^{\bullet}OH,tot}\ (\Sigma_i\ k_{Si}\ [S_i])^{-1}$.

Formation and quenching of singlet oxygen, $^1O_2$

Singlet oxygen is generated upon excitation of CDOM by sunlight. In this framework, the formation rate of $^1O_2$ can be assessed as follows:[3]

$$R_{^1O_2} = \int_{\lambda} \left[p^{\circ}(\lambda)\ \Phi_{^1O_2} (1-10^{-A_1(\lambda)}) \right]\ d\lambda \qquad (S16)$$

where $\Phi_{^1O_2} = 1.2\times10^{-3}$. Photogenerated $^1O_2$ is mainly quenched by collision with water, described by a first-order rate constant $k_q = 1\times10^5\ s^{-1}$.[8] On this basis, the steady-state $[^1O_2]$ is assessed as follows: $[^1O_{2\,w}]_w = R_{^1O_2}\ k_q^{-1}$.

Figure S2 reports the trends of the steady-state $[^{\bullet}OH]$ and $[^1O_2]$ assessed by means of the APEX software, following the above calculation procedures. Two different scenarios are assumed: (1) irradiation of a solution containing only CDOM, and (2) irradiation of a natural water sample. The steady-state $[^1O_2]$ would be equal at equal DOC in both cases, while $[^{\bullet}OH]$ would differ.

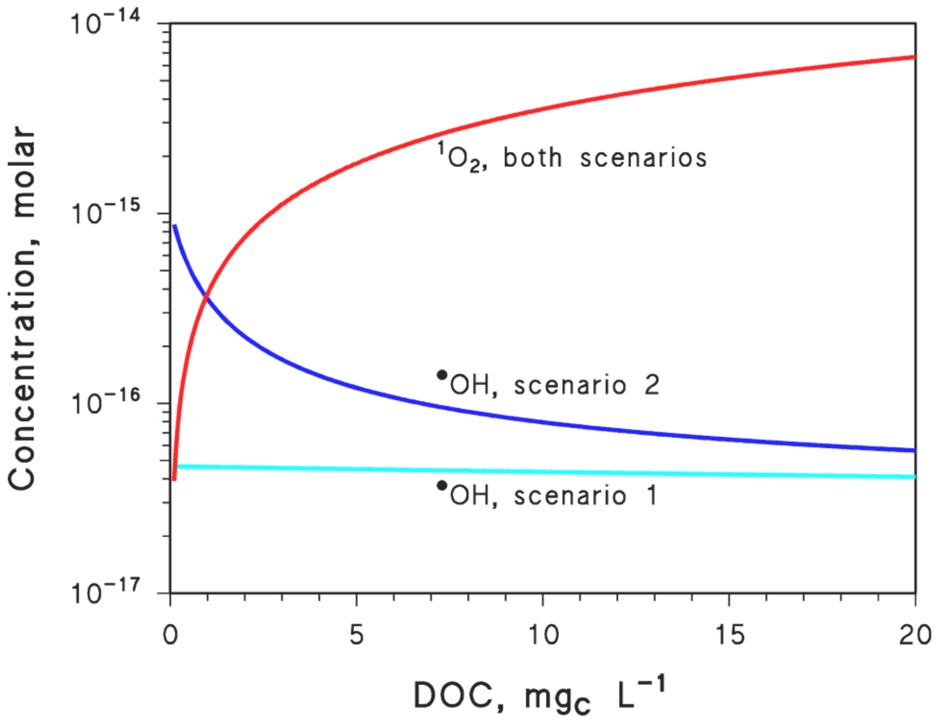

Figure S2. APEX modeling of [$^{\bullet}OH_w$]$_w$ and [$^1O_{2\ w}$]$_w$, as a function of the DOC. Water parameters are as follows: (scenario 1) 5 cm depth, absence of NO_3^-, NO_2^-, HCO_3^- and CO_3^{2-} as per irradiation experiments in the presence of humic substances alone; (scenario 2) 5 cm depth, 10^{-4} M NO_3^-, 10^{-6} M NO_2^-, 10^{-3} M HCO_3^-, and 10^{-5} M CO_3^{2-}, as per irradiation experiments in the presence of natural water samples. Sunlight irradiance: mid-latitude, average conditions in a fair-weather mid-summer day.

Table S1. Log_{10} K_{ow} values for various pollutants. When more than one value of Log_{10} K_{ow} was available in the literature for the same compound, the suggested Log_{10} K_{ow} value was that found most often. When this was not feasible, the average value is provided. Note that, for compounds undergoing acid-base equilibria, the Log_{10} K_{ow} value refers to the neutral form.

#	Pollutants	CAS	$Log_{10}K_{ow}$	Suggested Log_{10} K_{ow}	Use	Ref.
1	2,2',3,3',6,6'-Hexachlorobiphenyl	38411-22-2	6.70	6.70	Coolant and lubricant	9
2	2,2',4,4',6,6'-Hexachlorobiphenyl	33979-03-2	7.00	7.00	Coolant and lubricant	9
3	2,4,6-Tri-tert-butylphenol	732-26-3	6.39	6.39	Fuel additive	10
4	2,6-Diisopropylnaphthalene	24157-81-1	6.08	6.08	Solvent	10
5	4,4'-Ditertbutyldiphenylamine	4627-22-9	7.11	7.11	Ancillary ligand	10
6	Acesulfame K	55589-62-3	-1.36	-1.36	Artificial sweetener	10
7	Acetaminophen (paracetamol)	103-90-2	0.46	0.46	Analgesic and antipyretic	11,12
8	Amoxicillin	26787-78-0	0.87	0.87	Antibiotic	13
9	Aspirin	50-78-2	1.18	1.18	Anti-inflammatory drug	13
10	Atenolol	29122-68-7	0.16	0.16	Beta blocker	14
11	Atrazine	1912-24-9	2.61	2.61	Herbicide	14
12	Benzo[a]pyrene	50-32-8	6.11<br>6.20	6.15	Incomplete combustion product	9<br>9
13	Benzo[ghi]perylene	191-24-2	6.63<br>6.90	6.77	Incomplete combustion product	15<br>9
14	Benzophenone-3	131-57-7	3.52<br>3.79	3.79	UV filter	12<br>16,17
15	Benzophenone-4	4065-45-6	0.37	0.37	UV filter	18-20
16	Caffeine	58-08-2	-0.07<br>0.16	0.05	Stimulant	11,21<br>13,15
17	Carbamazepine	298-46-4	2.25<br>2.45<br>2.77	2.25	Anticonvulsant	13,15<br>22<br>23
18	Chlortoluron	15545-48-9	2.41	2.41	Herbicide	15
19	Clofenotane (DDT)	50-29-3	6.91	6.91	Insecticide	24
20	Clofibric acid	882-09-7	2.84<br>3.30	3.07	Lipid-lowering drug	13<br>12
21	Codeine	76-57-3	1.19<br>1.28<br>1.39	1.28	Analgesic	12<br>13,15<br>23
22	Decachlorobiphenyl	2051-24-3	8.26	8.26	Coolant and lubricant	9
23	Decane	124-18-5	5.01<br>6.25	5.63	Solvent, fuel and plasticiser	25<br>9
24	Dehydroabietine	5323-56-8	7.76	7.76	Antimicrobial and antifungal	10
25	Di(2-ethylhexyl) phthalate (DEHP)	117-81-7	7.50	7.50	Plasticizer	26
26	Diazepam (valium)	439-14-5	2.82	2.82	Anxiolytic	12,14

27	Diclofenac	15307-86-5	4.02<br>4.51	4.51	Anti-inflammatory drug	15<br>13,23,27
28	Diethyl phthalate (DEP)	84-66-2	2.38<br>2.42	2.40	Plasticizer	26<br>28
29	Diisobutyl phthalate (DiBP)	84-69-5	4.11	4.11	Plasticizer	26
30	Dimethomorph	110488-70-5	2.63<br>2.68	2.66	Fungicide	15<br>29
31	Dimethyl phthalate (DMP)	131-11-3	1.60	1.60	Insect repellant	28
32	Di-n-butyl phthalate (DnBP)	84-74-2	4.50	4.50	Plasticizer	28
33	Di-n-hexyl phthalate (DnHP).	84-75-3	6.30<br>6.82	6.56	Plasticizer	26<br>28
34	Diuron	330-54-1	2.68<br>2.8	2.8	Algicide and herbicide	30<br>15,30
35	Di-n-octyl phthalate	117-84-0	8.06	8.06	Adhesive and fragrance	26
36	Dodecahydrochrysene	1610-22-6	6.24	4.24		10
37	Eicosanoic acid	506-30-9	9.29	9.29	Opacifying agent and surfactant	9,15
38	Ethylhexylmethoxy cinnamate (EHMC)	5466-77-3	6.0	6.0	UV filter	31
39	Estrone	53-16-7	3.43	3.43	Steroid	13
40	Fenuron	101-42-8	0.98	0.98	Herbicide	15
41	Gemfibrozil	25812-30-0	4.77	4.77	Cholesterol regulator	13
42	Ibuprofen	15687-27-1	3.97	3.97	Anti-inflammatory drug	12,13,27
43	Imidacloprid	138261-41-3	-0.57<br>0.57	0.57	Insecticide	15<br>29,32
44	Lidocaine	137-58-6	2.26<br>2.45	2.36	Anesthetic	12<br>27
45	Metoprolol	51384-51-1	1.88<br>2.15	2.15	Beta blocker	15<br>14,23
46	Miconazole	22916-47-8	6.1	6.1	Antifungine	15
47	Naproxen	22204-53-1	3.18	3.18	Anti-inflammatory drug	13
48	Nicotine	54-11-5	1.17	1.17	Stimulant	21
49	Nitrobenzene	98-95-3	1.85	1.85	Chemical precursor and solvent	33
50	Octyldimethyl p-aminobenzoic acid (OD-PABA)	58817-05-3	6.15	6.15	Dermal penetration enhancer	20
51	Oxazepam	604-75-1	2.24	2.24	Anxiolytic and sedative	12,15,23
52	Prednisone	53-03-2	1.46<br>1.59	1.53	Anti-inflammatory drug	12<br>13
53	Progesterone	57-83-0	3.67<br>3.87	3.77	Steroid	13<br>12
54	Propanolol	525-66-6	3.48	3.48	Beta blocker	12,14,15,27
55	Sertraline	79617-96-2	5.29<br>5.50	5.43	Antidepressant	15<br>23

			5.51			14
56	Sulfadiazine	68-35-9	-0.09<br>-0.34	-0.09	Antibacterial	12,15<br>13
57	Tamoxifen	10540-29-1	7.1	7.1	Estrogen receptor modulator	15
58	Tonalide	21145-77-7	6.35	6.35	Fragrance	13
59	Triclosan	3380-34-5	5.34	5.34	Antibacterial and antifungal agent	13
60	Triisononyl trimellitate	53894-23-8	13.1	13.1	Plasticizer	10
61	Tris(2-ethyhexyl)phosphate (TEHP)	78-42-2	9.43	9.43	Plasticizer	34
62	Tris(4-isopropylphenyl)phosphate	2502-15-0	9.07	9.07	Flame retardant and plasticizer	10
63	Verapamil	52-53-9	3.79<br>5.00	3.79	Anti-hypertension drug	12,15<br>23
64	Warfarin	81-81-2	2.7<br>3.25	2.98	Anticoagulant	15<br>23

Appendix to Table S1. Partition and distribution coefficients

Compounds with a $pK_a < 8$ are also present in ionized form in natural waters (pH ~ 7).
Log P (log K_{ow}) refers to the undissociated form only. Therefore, we also report the distribution coefficients (log D) that that take into account the dissociation equilibria and are referred to the reported pH value.

Name	CAS #	pK_a	Log P[(i)]	LogD[(ii)] (pH)
Eicosanoic acid	506-30-9	~5[(iii)]	9.29[a–c]	6.25 (7.4)[d]
Di-n-octyl phthalate	117-84-0	high (?)	9.08[d]<br>8.10[c]<br>8.06[e]	8.53 (7.4)[d]
Clofenotane (DDT)	50-29-3	high (17.1?)[(iv)]	5.92[d]<br>6.91[c,f]<br>6.91 (7)[g]	6.22 (7.4)[d]
Decane	124-18-5	high (?)	6.07[d]<br>5.63[a,h]<br>5.01[c]	5.84 (7.4)[d]
Diclofenac	15307-86-5	4.15[c,i]	4.06[d]<br>4.51[c,i-k]	1.37 (7.4)[d]<br>0.96 (7?)[i]
Ibuprofen	15687-27-1	4.91[c,i]	3.97[c,i,k,l]<br>3.72[d]<br>3.68[m]<br>2.42[n]	1.25 (7?)[i]<br>0.45 (7.4)[d]<br>0.82 (7.4)[m]<br>1.28[n]
Diazepam (valium)	439-14-5	3.4[c]	2.91[d]<br>2.82[c,l,o]	2.92 (7.4)[d]
Aspirin	50-78-2	3.47[c]	1.19[d]<br>1.27[p]<br>1.18[c,o]	-1.69 (7.4)[d]<br>-2.7 (7.4)[p]
Acetaminophen (paracetamol)	103-90-2	9.38[c]	0.46[c,i,l]<br>0.34[d]	0.90 (7?)[i]<br>0.4 (7.4)[d]
Sulfadiazine	68-35-9	6.36[c]	-0.34[i]<br>-0.12[d]<br>-0.09[c,l,q]	-0.23 (7?)[i]<br>-0.79 (7.4)[d]

Notes:
(i) partition coefficient (un-ionized species only)
(ii) distribution coefficient (ionized and un-ionized species)
(iii) estimated value based on pKa data of another fatty acid [r]
(iv) Bo-Long Poh [**Australian Journal of Chemistry**, **1979**, vol. 32, p. 429,431]

References:

(a) Octanol-Water Partition Coefficients. In *CRC Handbook of Chemistry and Physics*; Rumble, J. R., Ed.; CRC Press/Taylor & Francis: Boca Raton, FL.
(b) Hansch, C.; Leo, A.; Hoekman, D. *Exploring QSAR: Hydrophobic, Electronic, and Steric Constants*; ACS Professional Reference Book; American Chemical Society: Washington, DC, 1995.
(c) PubChem. *PubChem*. https://pubchem.ncbi.nlm.nih.gov/ (accessed 2025-04-11).
(d) *ChemSpider: Search and Share Chemistry - Homepage*. https://www.chemspider.com/ (accessed 2025-04-11).
(e) Staples, C. A.; Peterson, D. R.; Parkerton, T. F.; Adams, W. J. The Environmental Fate of Phthalate Esters: A Literature Review. *Chemosphere* **1997**, *35* (4), 667–749. https://doi.org/10.1016/S0045-6535(97)00195-1.
(f) Finizio, A.; Vighi, M.; Sandroni, D. Determination of N-Octanol/Water Partition Coefficient (Kow) of Pesticide Critical Review and Comparison of Methods. *Chemosphere* **1997**, *34* (1), 131–161. https://doi.org/10.1016/S0045-6535(96)00355-4.
(g) Palma, D. C. A.; Lourencetti, C.; Uecker, M. E.; Mello, P. R. B.; Pignati, W. A.; Dores, E. F. G. C. Simultaneous Determination of Different Classes of Pesticides in Breast Milk by Solid-Phase Dispersion and GC/ECD. *J. Braz. Chem. Soc.* **2014**, *25*, 1419–1430. https://doi.org/10.5935/0103-5053.20140124.
(h) Coates, Michael.; Connell, D. W.; Barron, D. M. Aqueous Solubility and Octan-1-Ol-Water Partition Coefficients of Aliphatic Hydrocarbons. *Environ. Sci. Technol.* **1985**, *19* (7), 628–632. https://doi.org/10.1021/es00137a008.
(i) Ilyas, H.; Masih, I.; van Hullebusch, E. D. Prediction of the Removal Efficiency of Emerging Organic Contaminants in Constructed Wetlands Based on Their Physicochemical Properties. *J. Environ. Manage.* **2021**, *294*, 112916. https://doi.org/10.1016/j.jenvman.2021.112916.
(j) Grabic, R.; Ivanová, L.; Kodešová, R.; Grabicová, K.; Vojs Staňová, A.; Imreová, Z.; Drtil, M.; Bodík, I. Desorption of Pharmaceuticals and Illicit Drugs from Different Stabilized Sludge Types across pH. *Water Res.* **2022**, *220*, 118651. https://doi.org/10.1016/j.watres.2022.118651.
(k) Avdeef, A.; Box, K. J.; Comer, J. E. A.; Hibbert, C.; Tam, K. Y. pH-Metric logP 10. Determination of Liposomal Membrane-Water Partition Coefficients of Lonizable Drugs. *Pharm. Res.* **1998**, *15* (2), 209–215. https://doi.org/10.1023/A:1011954332221.
(l) Pratiwi, R.; Mahmudah, D. I. A.; Saptarini, N. M.; Suherman, M.; Megantara, S. COMPARISON OF PARTITION COEFFICIENT (LOG P) OF DRUGS: COMPUTATIONAL AND EXPERIMENTAL DATA STUDY. *Int. J. Appl. Pharm.* **2023**, *15* (Special Issue 2), 155–162. https://doi.org/10.22159/ijap.2023.v15s2.29.
(m) Sodano, F.; Cristiano, C.; Rolando, B.; Marini, E.; Lazzarato, L.; Cuozzo, M.; Albrizio, S.; Russo, R.; Rimoli, M. G. Galactosylated Prodrugs: A Strategy to Improve the Profile of Nonsteroidal Anti-Inflammatory Drugs. *Pharmaceuticals* **2022**, *15* (5), 552. https://doi.org/10.3390/ph15050552.
(n) Janus, E.; Ossowicz, P.; Klebeko, J.; Nowak, A.; Duchnik, W.; Kucharski, Ł.; Klimowicz, A. Enhancement of Ibuprofen Solubility and Skin Permeation by Conjugation with L-Valine Alkyl Esters. *RSC Adv. 10* (13), 7570–7584. https://doi.org/10.1039/d0ra00100g.
(o) Hwang, Y.; Sohn, J.-T. Effect of Lipid Emulsion on Neuropsychiatric Drug-Induced Toxicity: A Narrative Review. *Medicine (Baltimore)* **2024**, *103* (11), e37612. https://doi.org/10.1097/MD.0000000000037612.
(p) Martin-Martin, A.; Rivera-Dictter, A.; Muñoz-Uribe, M.; López-Contreras, F.; Pérez-Laines, J.; Molina-Berríos, A.; López-Muñoz, R. Reconsidering the Role of Cyclooxygenase Inhibition in the Chemotherapeutic Value of NO-Releasing Aspirins for Lung Cancer. *Molecules* **2019**, *24* (10), 1924. https://doi.org/10.3390/molecules24101924.
(q) Robinson, R. F. A.; Mills, G. A.; Grabic, R.; Bořík, A.; Fones, G. R. Quantification and Risk Assessment of Polar Organic Contaminants in Two Chalk Streams in Hampshire, UK Using the Chemcatcher Passive Sampler. *Sci. Total Environ.* **2024**, *939*, 173316. https://doi.org/10.1016/j.scitotenv.2024.173316.
(r) Davis, M. M.; Paabo, M. Comparative Strengths of Aliphatic Acids and Some Other Carboxylic Acids in Benzene at 25°. *J. Org. Chem.* **1966**, *31* (6), 1804–1810. https://doi.org/10.1021/jo01344a032.

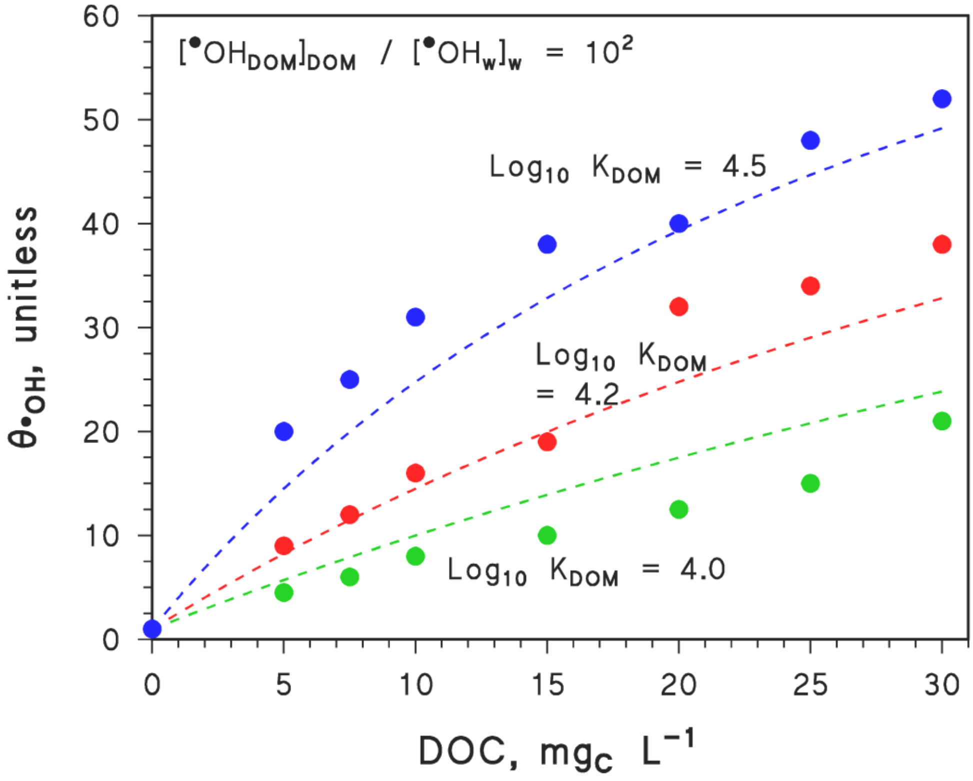

Figure S3. Comparison between experimental data [1] and model predictions for the $^{\bullet}$OH reaction trend in the case of hydrophobic pollutants ($C_{11}H_{18}Cl_6$ with Log_{10} K_{DOM} = 4.0, $C_{15}H_{26}Cl_6$ with Log_{10} K_{DOM} = 4.2, $C_{19}H_{33}Cl_7$ with Log_{10} K_{DOM} = 4.5). In the case of the experimental data, the values of $\theta_{^{\bullet}OH}$ were derived from the reported $[^{\bullet}OH]_{app}$ values of Yan et al.[1]. There is reasonable agreement between the model and the experimental data when assuming $[^{\bullet}OH_{DOM}]_{DOM}$ $([^{\bullet}OH_w]_w)^{-1} = 10^2$.

$$\theta_{^{\bullet}OH} = \left(\frac{1}{[^{\bullet}OH_w]_w}\right)_{DOC\to 0} \frac{[^{\bullet}OH_w]_w + 2.6\cdot 10^{-7}\, K_{ow}\, DOC\, [^{\bullet}OH_{DOM}]_{DOM}}{1 + 2.6\cdot 10^{-7}\, K_{ow}\, DOC} \qquad (S17)$$

Assume that $[^{\bullet}OH_w]_w \sim ([^{\bullet}OH_w]_w)_{DOC\to 0}$, which is reasonable in a system that contains only CDOM (see **Figure S2**, scenario 1). In this case, **equation (S18)** becomes:

$$\theta_{^{\bullet}OH} = \frac{1 + 2.6\cdot 10^{-7}\, K_{ow}\, DOC\, [^{\bullet}OH_{DOM}]_{DOM} / [^{\bullet}OH_w]_w}{1 + 2.6\cdot 10^{-7}\, K_{ow}\, DOC} \qquad (S18)$$

Fit of the experimental data was carried out using **equation (S18)**, with $\Re = [^{\bullet}OH_{DOM}]_{DOM} / [^{\bullet}OH_w]_w$ as the fit variable.

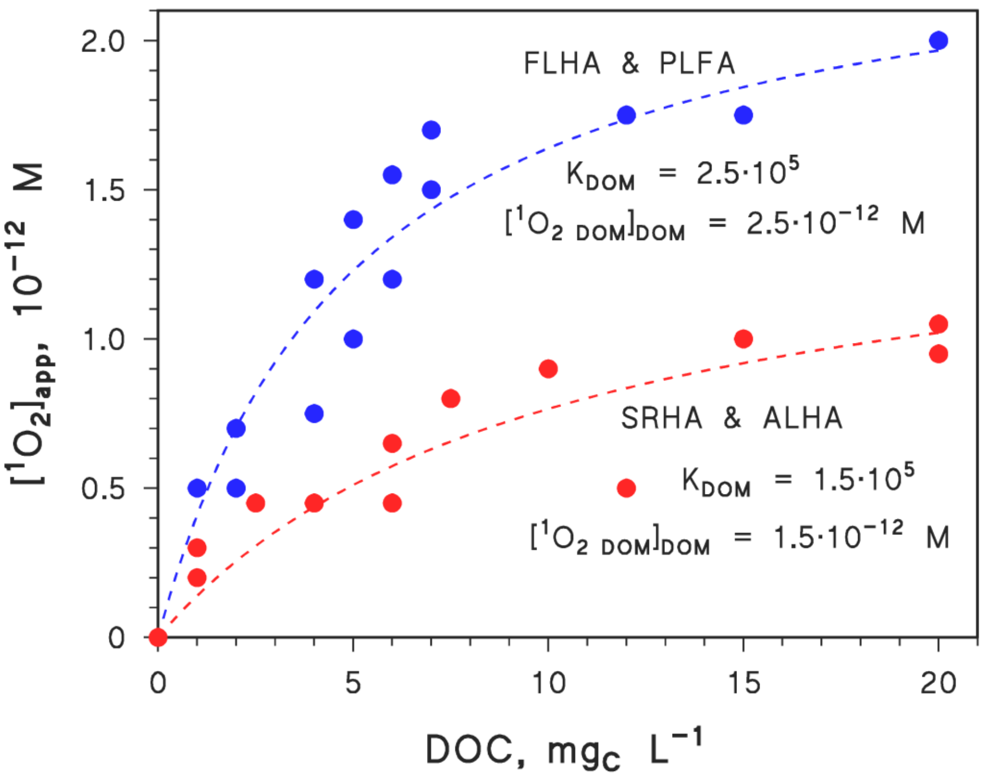

Figure S4. Comparison between experimental data [35] and model predictions **(equation S(20))** for the $^1O_2$ reaction trend in the case of Fluka Humic Acid (FLHA), Pony Lake Fulvic Acid (PLFA), Suwannee River Humic Acid (SRHA), and Aldrich Humic Acid (ALHA).

$$[^1O_2]_{app} = \frac{[^1O_{2\ w}]_w + 10^{-6}\, K_{DOM}\, DOC\ [^1O_{2\ DOM}]_{DOM}}{1 + 10^{-6}\ K_{DOM}\, DOC} \qquad (S19)$$

Assuming $[^1O_{2\ w}]_w \ll 10^{-6}\, K_{DOM}\, DOC\ [^1O_{2\ DOM}]_{DOM}$, **equation (S19)** gets transformed as follows:

$$[^1O_2]_{app} = \frac{1.3\times10^{-12}\, K_{DOM}\, DOC^2[^1O_{2\ DOM}]_{DOM}}{1 + 10^{-6}\, K_{DOM}\, DOC} \qquad (S20)$$

Equation (S20) was used to fit the experimental data and derive the values of $[^1O_{2\ DOM}]_{DOM}$. Their average is $[^1O_{2\ DOM}]_{DOM} \sim 2\times10^{-12}$ M. On this basis, considering that $[^1O_{2\ w}]_w$ would vary between 4×10^{-17} and 7×10^{-15} M and that $10^{-6}\, K_{DOM}\, DOC \sim 1$, the initial assumption that $[^1O_{2\ w}]_w \ll 10^{-6}\, K_{DOM}\, DOC\ [^1O_{2\ DOM}]_{DOM}$ is easily satisfied.

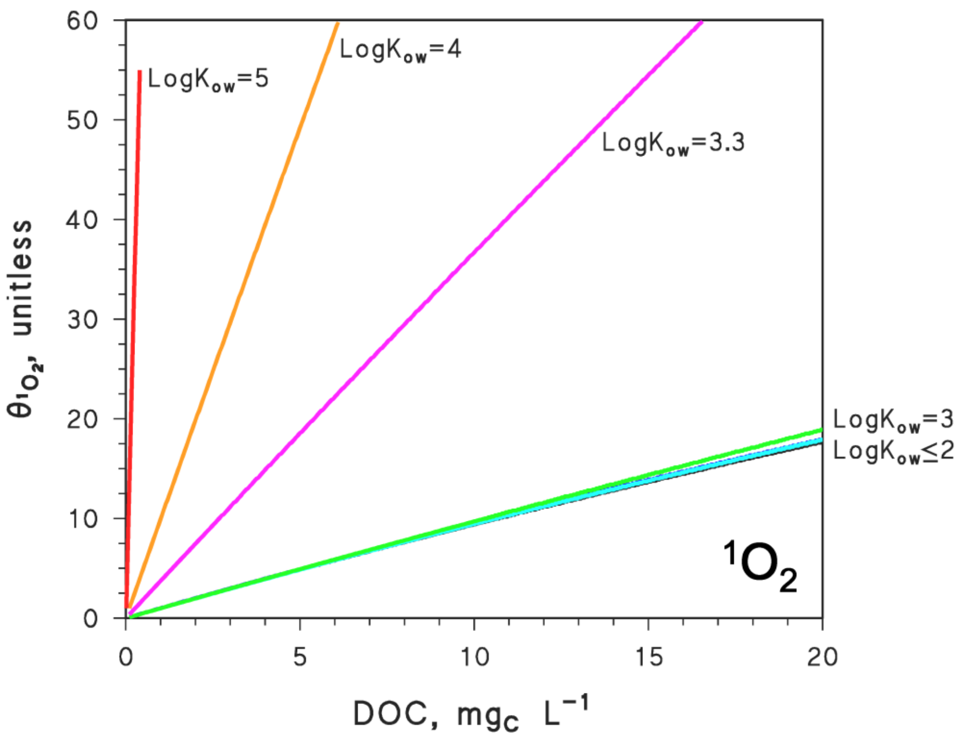

Figure S5. Trends of $\theta_{^1O_2}$ *vs*. DOC, for different values of $\text{Log}_{10} K_{ow}$. The calculation of $\theta_{^1O_2}$ is based on **equation (S21)** (see below, it is equivalent to equation (6) for $^{\bullet}$OH in the main text), and $\theta_{^1O_2}$ was normalized to the value obtained with $K_{ow} \rightarrow 0$ and DOC = 1 mg$_C$ L^{-1} (arbitrary choice). Normalization for DOC → 0 as in the case of $^{\bullet}$OH is made problematic by the fact that, when DOC → 0, it is $[^1O_{2\ w}]_w \rightarrow 0$ as well. Water conditions: 5 cm depth, absence of NO_3^-, NO_2^-, HCO_3^-, and CO_3^{2-} as per irradiation experiments in the presence of humic substances alone. Note, however, that $[^1O_{2\ w}]_w$ is unaffected by nitrate, nitrite, or inorganic carbon.

$$\theta_{^1O_2} = \left(\frac{1}{[^1O_{2\ w}]_w}\right)_{DOC\rightarrow 1} \frac{[^1O_{2\ w}]_w + 1.3\times 10^{-12}\, K_{\mathrm{DOM}}\, \mathrm{DOC}^2 [^1O_{2\ \mathrm{DOM}}]_{\mathrm{DOM}}}{1 + 10^{-6}\, K_{\mathrm{DOM}}\, \mathrm{DOC}} \qquad \text{(S21)}$$

References

(1) Yan, S.; Sun, J.; Sha, H.; Li, Q.; Nie, J.; Zou, J.; Chu, C.; Song, W. Microheterogeneous Distribution of Hydroxyl Radicals in Illuminated Dissolved Organic Matter Solutions. *Environ. Sci. Technol.* **2021**, *55* (15), 10524–10533. https://doi.org/10.1021/acs.est.1c03346.

(2) Poerschmann, J.; Kopinke, F.-D. Sorption of Very Hydrophobic Organic Compounds (VHOCs) on Dissolved Humic Organic Matter (DOM). 2. Measurement of Sorption and Application of a Flory−Huggins Concept To Interpret the Data. *Environ. Sci. Technol.* **2001**, *35* (6), 1142–1148. https://doi.org/10.1021/es0017615.

(3) Vione, D. A Critical View of the Application of the APEX Software (Aqueous Photochemistry of Environmentally-Occurring Xenobiotics) to Predict Photoreaction Kinetics in Surface Freshwaters. *Molecules* **2020**, *25*, 1. https://doi.org/10.3390/molecules25010009.

(4) Vione, D.; Das, R.; Rubertelli, F.; Maurino, V.; Minero, C.; Barbati, S.; Chiron, S. Modelling the occurrence and reactivity of hydroxyl radicals in surface waters: Implications for the fate of selected pesticides. *Intern. J. Environ. Anal. Chem.* **2010**, *90*, 258-273. https://doi.org/10.1080/03067310902894218.

(5) Page, S. E.; Arnold, W. A.; McNeill, K. Assessing the contribution of free hydroxyl radical in organic matter-sensitized photohydroxylation reactions. *Environ. Sci. Technol.* **2011**, *45*, 2818-2825. https://doi.org/10.1021/es2000694.

(6) Mostofa, K. M. G.; Sakugawa, H. Spatial and temporal variations and factors controlling the concentrations of hydrogen peroxide and organic peroxides in rivers. *Environ. Chem.* **2009**, *6*, 524-534. https://doi.org/10.1071/EN09070_AC.

(7) Frank, R.; Klöpffer, W. Spectral solar photon irradiance in Central Europe and the adjacent North Sea. *Chemosphere* **1988**, *17*, 985-994. https://doi.org/10.1016/0045-6535(88)90069-0.

(8) Appiani, E.; Ossola, R.; Latch, D. E.; Erickson, P. R.; McNeill, K. Aqueous singlet oxygen reaction kinetics of furfuryl alcohol: effect of temperature, pH, and salt content. *Environ. Sci.: Processes Impacts* **2017**, *19*, 507-516. http://dx.doi.org/10.1039/C6EM00646A.

(9) Octanol-Water Partition Coefficients. In *CRC Handbook of Chemistry and Physics*; Rumble, J. R., Ed.; CRC Press/Taylor & Francis: Boca Raton, FL.

(10) Vione, D.; Arey, J. S.; Parkerton, T. F.; Redman, A. D. Direct and Indirect Photodegradation in Aquatic Systems Mitigates Photosensitized Toxicity in Screening-Level Substance Risk Assessments of Selected Petrochemical Structures. *Water Res.* **2024**, *257*, 121677. https://doi.org/10.1016/j.watres.2024.121677.

(11) Stachowiak, W.; Rzemieniecki, T.; Klejdysz, T.; Pernak, J.; Niemczak, M. “Sweet” Ionic Liquids Comprising the Acesulfame Anion – Synthesis, Physicochemical Properties and Antifeedant Activity towards Stored Product Insects. *New J. Chem.* **2020**, *44* (17), 7017–7028. https://doi.org/10.1039/C9NJ06005G.

(12) Pratiwi, R.; Mahmudah, D. I. A.; Saptarini, N. M.; Suherman, M.; Megantara, S. Comparison of partition coefficient (Log P) of drugs: Computational and experimental data study. *Int. J. Appl. Pharm.* **2023**, *15* (Special Issue 2), 155–162. https://doi.org/10.22159/ijap.2023.v15s2.29.

(13) Ilyas, H.; Masih, I.; van Hullebusch, E. D. Prediction of the Removal Efficiency of Emerging Organic Contaminants in Constructed Wetlands Based on Their Physicochemical Properties. *J. Environ. Manage.* **2021**, *294*, 112916. https://doi.org/10.1016/j.jenvman.2021.112916.

(14) Hwang, Y.; Sohn, J.-T. Effect of Lipid Emulsion on Neuropsychiatric Drug-Induced Toxicity: A Narrative Review. *Medicine (Baltimore)* **2024**, *103* (11), e37612. https://doi.org/10.1097/MD.0000000000037612.

(15) Robinson, R. F. A.; Mills, G. A.; Grabic, R.; Bořík, A.; Fones, G. R. Quantification and Risk Assessment of Polar Organic Contaminants in Two Chalk Streams in Hampshire, UK Using the Chemcatcher Passive Sampler. *Sci. Total Environ.* **2024**, *939*, 173316. https://doi.org/10.1016/j.scitotenv.2024.173316.

(16) Hansch, C.; Leo, A.; Hoekman, D. *Exploring QSAR: Hydrophobic, Electronic, and Steric Constants*; ACS Professional Reference Book; American Chemical Society: Washington, DC, 1995.

(17) Wu, C.; Huang, X.; Lin, J.; Liu, J. Occurrence and Fate of Selected Endocrine-Disrupting Chemicals in Water and Sediment from an Urban Lake. *Arch. Environ. Contam. Toxicol.* **2015**, *68* (2), 225–236. https://doi.org/10.1007/s00244-014-0087-6.

(18) Jou-Claus, S.; Rodríguez-Escales, P.; Martínez-Landa, L.; Diaz-Cruz, M. S.; Carrera, J.; Sunyer-Caldú, A.; Quintana, G.; Valhondo, C. Assessing the Fate of Benzophenone-Type UV Filters and Transformation Products during Soil Aquifer Treatment: The Biofilm Compartment as Bioaccumulator and Biodegrader in Porous Media. *Environ. Sci. Technol.* **2024**, *58* (12), 5472–5482. https://doi.org/10.1021/acs.est.3c08465.

(19) Molins-Delgado, D.; Díaz-Cruz, M. S.; Barceló, D. Ecological Risk Assessment Associated to the Removal of Endocrine-Disrupting Parabens and Benzophenone-4 in Wastewater Treatment. *J. Hazard. Mater.* **2016**, *310*, 143–151. https://doi.org/10.1016/j.jhazmat.2016.02.030.

(20) Vidal-Liñán, L.; Villaverde-de-Sáa, E.; Rodil, R.; Quintana, J. B.; Beiras, R. Bioaccumulation of UV Filters in *Mytilus Galloprovincialis* Mussel. *Chemosphere* **2018**, *190*, 267–271. https://doi.org/10.1016/j.chemosphere.2017.09.144.

(21) Sousa, R. de C. S.; Pereira, M. M.; Freire, M. G.; Coutinho, J. A. P. Evaluation of the Effect of Ionic Liquids as Adjuvants in Polymer-Based Aqueous Biphasic Systems Using Biomolecules as Molecular Probes. *Sep. Purif. Technol.* **2018**, *196*, 244–253. https://doi.org/10.1016/j.seppur.2017.07.018.

(22) Dal Pozzo, A.; Donzelli, G.; Rodriquez, L.; Tajana, A. "In Vitro" Model for the Evaluation of Drug Distribution and Plasma Protein-Binding Relationships. *Int. J. Pharm.* **1989**, *50* (2), 97–101. https://doi.org/10.1016/0378-5173(89)90133-6.

(23) Grabic, R.; Ivanová, L.; Kodešová, R.; Grabicová, K.; Vojs Staňová, A.; Imreová, Z.; Drtil, M.; Bodík, I. Desorption of Pharmaceuticals and Illicit Drugs from Different Stabilized Sludge Types across pH. *Water Res.* **2022**, *220*, 118651. https://doi.org/10.1016/j.watres.2022.118651.

(24) Finizio, A.; Vighi, M.; Sandroni, D. Determination of N-Octanol/Water Partition Coefficient (Kow) of Pesticide Critical Review and Comparison of Methods. *Chemosphere* **1997**, *34* (1), 131–161. https://doi.org/10.1016/S0045-6535(96)00355-4.

(25) Coates, Michael.; Connell, D. W.; Barron, D. M. Aqueous Solubility and Octan-1-Ol-Water Partition Coefficients of Aliphatic Hydrocarbons. *Environ. Sci. Technol.* **1985**, *19* (7), 628–632. https://doi.org/10.1021/es00137a008.

(26) Staples, C. A.; Peterson, D. R.; Parkerton, T. F.; Adams, W. J. The Environmental Fate of Phthalate Esters: A Literature Review. *Chemosphere* **1997**, *35* (4), 667–749. https://doi.org/10.1016/S0045-6535(97)00195-1.

(27) Avdeef, A.; Box, K. J.; Comer, J. E. A.; Hibbert, C.; Tam, K. Y. pH-Metric logP 10. Determination of Liposomal Membrane-Water Partition Coefficients of Lonizable Drugs. *Pharm. Res.* **1998**, *15* (2), 209–215. https://doi.org/10.1023/A:1011954332221.

(28) Ellington, J. J. Octanol/Water Partition Coefficients and Water Solubilities of Phthalate Esters. *J. Chem. Eng. Data* **1999**, *44* (6), 1414–1418. https://doi.org/10.1021/je990149u.

(29) Cui, K.; Wang, J.; Guan, S.; Liang, J.; Fang, L.; Ding, R.; Li, T.; Dong, Z.; Ma, G.; Wu, X.; Zheng, Y. Residue Changes, Degradation, Processing Factors and Their Relation between Physicochemical Properties of Pesticides in Peanuts during Multiproduct Processing. *Food Chem.* **2024**, *452*, 139535. https://doi.org/10.1016/j.foodchem.2024.139535.

(30) Cui, Y. T.; Teo, S. L. M.; Leong, W.; Chai, C. L. L. Searching for "Environmentally-Benign" Antifouling Biocides. *Int. J. Mol. Sci.* **2014**, *15* (6), 9255–9284. https://doi.org/10.3390/ijms15069255.

(31) Balmer, M. E.; Buser, H.-R.; Müller, M. D.; Poiger, T. Occurrence of Some Organic UV Filters in Wastewater, in Surface Waters, and in Fish from Swiss Lakes. *Environ. Sci. Technol.* **2005**, *39* (4), 953–962. https://doi.org/10.1021/es040055r.

(32) Wang, M.; Tian, Q.; Li, H.; Dai, L.; Wan, Y.; Wang, M.; Han, B.; Huang, H.; Zhang, Y.; Chen, J. Visualization and Metabolome for the Migration and Distribution Behavior of Pesticides Residue in After-Ripening of Banana. *J. Hazard. Mater.* **2023**, *446*, 130665. https://doi.org/10.1016/j.jhazmat.2022.130665.

(33) Andersson, J. T.; Schräder, W. A Method for Measuring 1-Octanol−Water Partition Coefficients. *Anal. Chem.* **1999**, *71* (16), 3610–3614. https://doi.org/10.1021/ac9902291.

(34) Wang, Y.; Gao, F.; Xu, Y.; Rodgers, T. F. M.; Tan, F. Field Study on the Uptake Pathways and Their Contributions to the Accumulation of Organophosphate Esters, Phthalates, and Polycyclic Aromatic Hydrocarbons in Upland Rice. *Sci. Total Environ.* **2024**, *946*, 174205. https://doi.org/10.1016/j.scitotenv.2024.174205.

(35) Grandbois, M.; Latch, D. E.; McNeill, K. Microheterogeneous Concentrations of Singlet Oxygen in Natural Organic Matter Isolate Solutions. *Environ. Sci. Technol.* **2008**, *42* (24), 9184–9190. https://doi.org/10.1021/es8017094.
